# Gastroenteropancreatic neuroendocrine neoplasms: current development, challenges, and clinical perspectives

**DOI:** 10.1186/s40779-024-00535-6

**Published:** 2024-06-04

**Authors:** Xian-Bin Zhang, Yi-Bao Fan, Rui Jing, Mikiyas Amare Getu, Wan-Ying Chen, Wei Zhang, Hong-Xia Dong, Tikam Chand Dakal, Akhtar Hayat, Hua-Jun Cai, Milad Ashrafizadeh, A. M. Abd El-Aty, Ahmet Hacimuftuoglu, Peng Liu, Tian-Feng Li, Gautam Sethi, Kwang Seok Ahn, Yavuz Nuri Ertas, Min-Jiang Chen, Jian-Song Ji, Li Ma, Peng Gong

**Affiliations:** 1 Department of General SurgeryInstitute of Precision Diagnosis and Treatment of Digestive System Tumors and Guangdong Provincial Key Laboratory of Chinese Medicine Ingredients and Gut Microbiomics, Carson International Cancer Center, Shenzhen University General Hospital, Shenzhen University, Shenzhen, Guangdong 518055 China; 2 School of Pharmacy, Shenzhen University Medical School, Shenzhen University, Shenzhen, Guangdong 518060 China; 3 Department of Radiology, Second Hospital of Shandong University, Jinan, Shandong 250000 China; 4 Department of Gastroenterology, General Hospital of Chinese PLA, Beijing, 100853 China; 5 Department of Biotechnology, Mohanlal Sukhadia University, Udaipur, Rajasthan 313001 India; 6 Interdisciplinary Research Centre in Biomedical Materials (IRCBM), COMSATS University Islamabad, Lahore Campus, Lahore, 54000 Pakistan; 7 Department of Pharmacology, Faculty of Veterinary Medicine, Cairo University, Giza, 12211 Egypt; 8 Department of Medical Pharmacology, Medical Faculty, Ataturk University, Erzurum, 25240 Turkey; 9 Reproductive Medicine Center, Shenzhen Maternity & Child Healthcare Hospital, Southern Medical University, Shenzhen, Guangdong 518055 China; 10 Department of Pharmacology, Yong Loo Lin School of Medicine, National University of Singapore, Singapore, 117600 Singapore; 11 Department of Science in Korean Medicine, Kyung Hee University, Seoul, 02447 Republic of Korea; 12 ERNAM-Nanotechnology Research and Application Center, Erciyes University, Kayseri, 38039 Türkiye; 13 Department of Biomedical Engineering, Erciyes University, Kayseri, 38280 Türkiye; 14 UNAM−National Nanotechnology Research Center, Bilkent University, Ankara, 06800 Türkiye; 15 Key Laboratory of Imaging Diagnosis and Minimally Invasive Intervention Research, Fifth Affiliated Hospital of Wenzhou Medical University, Lishui, Zhejiang 323000 China; 16 Department of Epidemiology, Dalian Medical University, Dalian, Liaoning 116044 China

**Keywords:** Gastroenteropancreatic neuroendocrine neoplasms (GEP-NENs), Neuroendocrine neoplasms (NENs), Diagnosis, Chemotherapy, Immunotherapy

## Abstract

Neuroendocrine neoplasms (NENs) are highly heterogeneous and potentially malignant tumors arising from secretory cells of the neuroendocrine system. Gastroenteropancreatic neuroendocrine neoplasms (GEP-NENs) are the most common subtype of NENs. Historically, GEP-NENs have been regarded as infrequent and slow-growing malignancies; however, recent data have demonstrated that the worldwide prevalence and incidence of GEP-NENs have increased exponentially over the last three decades. In addition, an increasing number of studies have proven that GEP-NENs result in a limited life expectancy. These findings suggested that the natural biology of GEP-NENs is more aggressive than commonly assumed. Therefore, there is an urgent need for advanced researches focusing on the diagnosis and management of patients with GEP-NENs. In this review, we have summarized the limitations and recent advancements in our comprehension of the epidemiology, clinical presentations, pathology, molecular biology, diagnosis, and treatment of GEP-NETs to identify factors contributing to delays in diagnosis and timely treatment of these patients.

## Background

Neuroendocrine neoplasms (NENs) originate from specialized secretory cells within the diffuse neuroendocrine system, with approximately two-thirds manifesting in the gastroenteropancreatic system [1–3]. Although they are uncommon, gastroenteropancreatic neuroendocrine neoplasms (GEP-NENs) have significantly increased in global prevalence and incidence over the past three decades. This class of tumors exhibits a wide range of diversity and complexity, varying from indolent to aggressive. Furthermore, due to their rarity, there is a lack of understanding regarding diagnosis and treatment among clinicians, patients, and the general public. Genetic variations further complicate our understanding of disease biology and hinder the development of targeted therapies [4, 5]. Consequently, effective strategies for diagnosing and treating these tumors are lacking. Moreover, the prognosis of GEP-NENs is uncertain due to the absence of reliable prognostic markers, often leading to delayed diagnoses at advanced stages [4–7]. Therefore, it is crucial to conduct advanced research and develop innovative treatment approaches to improve the diagnosis and management of GEP-NENs. This review provides a comprehensive overview of the epidemiology, clinical presentation, diagnosis, management, challenges, and future perspectives of GEP-NENs. The insights derived from this review aim to inform and guide the development of diagnostic and therapeutic strategies for these rare neoplasms.

## Epidemiology

NENs are generally considered rare diseases. However, recent reports have shown a remarkable increase in the incidence of GEP-NENs worldwide [8, 9]. An analysis according to National Cancer Data in the United States indicated that the age-adjusted incidence of GEP-NENs increased from 1.05 per 100,000 persons in 1975 to 5.45 per 100,000 persons in 2015, which is quite alarming [2]. There are also similar trends in European populations [10–14]. Korse et al. [10] evaluated epidemiological data from the Netherlands and reported that the age-standardized incidence of GEP-NENs increased from 2.1 per 100,000 persons in 1990 to 4.9 per 100,000 persons in 2010. Despite the lower incidence in Asia compared with that in the United States and Europe [15, 16], it also increased from 0.244 per 100,000 in 1996 to 3.162 per 100,000 in 2015 [16]. Several factors may account for the lower incidence of GEP-NENs in Asia. First, due to the variations in healthcare infrastructure and resources between Asia and the United States, access to and utilization of services for early screening of cancer are more common in North America than in Asia. Second, detection technologies, including multiphase contrast-enhanced computed tomography (CT), magnetic resonance imaging (MRI), nuclear medicine, and biomarker assessment, are more readily available in the United States than in Asia. This may hinder early detection and timely intervention in Asian patients. Third, the data gathered from cancer registries in Asia could contribute to the lower incidence of NENs, given that the registries are not yet fully established [5]. Interestingly, several studies have shown that sex plays an essential role in the incidence and prognosis of GEP-NENs. Leoncini et al. [17] systematically analyzed 11 studies involving 72,048 patients and reported that males exhibit a greater incidence of high-grade NENs. This finding was supported by other studies, which also demonstrated that males are more likely to develop malignant NENs [1, 18, 19]. Differences in dietary habits may contribute to differences in the incidence of GEP-NENs between males and females [20, 21]. Usually, males tend to have a preference for consuming significant quantities of red meat, which can increase the risk of cancer development. In contrast, females are more inclined to consume fruits and vegetables, which are rich in antioxidants and other beneficial nutrients that may have the potential to reduce the risk of GEP-NENs [20, 21]. Furthermore, there are notable variations in hormone levels between males and females, with certain female hormones potentially influencing the occurrence of GEP-NENs. This hypothesis is substantiated by the consensus of multiple experts and research findings [22, 23]. However, the impact of sex on the incidence and response to specific drugs remains poorly understood. Therefore, further research is necessary to address the following questions: do female hormones impact the occurrence of GEP-NENs and treatment efficacy?

## Clinical presentations

The clinical manifestations of GEP-NENs vary widely, primarily depending on the tumor’s capacity to store and secrete biologically active hormones. Certain hormones are associated with specific clinical syndromes, while others are not. Based on these findings, the clinical presentations of NENs can be categorized into functional and non-functional manifestations, as outlined in Table 1 [3, 9, 16]. Typically, functional GEP-NENs secrete hormones, resulting in the development of a clinical condition characterized by hormone overproduction. For example, insulinomas are insulin-secreting tumors associated with hypoglycemia symptoms, including palpitations, diaphoresis, and altered mental status. Gastrinomas secrete gastrin, leading to excessive acid production and esophagitis, which can cause severe peptic ulcer disease, gastroesophageal reflux disease, and chronic diarrhea. Glucagonomas are characterized by symptoms such as necrolytic migratory erythema, hyperglycemia, diabetes mellitus, weight loss, and diarrhea. VIPomas autonomously secrete vasoactive intestinal polypeptide, resulting in watery diarrhea, hypokalemia, and achlorohydria. Classic carcinoid syndrome manifests as flushing, wheezing, and diarrhea due to the hypersecretion of serotonin and other vasoactive amines, such as histamine, tachykinins, and prostaglandins. However, non-functional GEP-NENs do not exhibit any hormone-related clinical symptoms; instead, their primary symptoms are tumor growth and metastasis. Thus, the clinical presentation of non-functional NENs can be asymptomatic or accompanied by abdominal pain, weight loss, or fatigue.

**Table 1:** Comparison of functional and non-functional NENs

Items	Functional NENs	Non-functional NENs
Hormones	Secrete bioactive substances; Clinical manifestations are related to the specific hormones’ secretion	Do not secrete bioactive substances; They may produce hormones, but the levels are usually not high enough to cause noticeable symptoms
Sign and symptoms	It depends on the hormone being produced; Examples and associated symptoms: Insulinoma: hypoglycemia, palpitations, diaphoresis, and confusion; Gastrinoma: esophagitis, peptic ulcer disease, gastroesophageal reflux disease, and diarrhea; Carcinoid syndrome: flushing, diarrhea, secretion of vasoactive mines, and bronchospasm	Related to the tumor size, location, and invasion of nearby structures; Symptoms may include abdominal pain, weight loss, fatigue, and other non-specific symptoms
Diagnostic methods	Hormone levels were measured and imaging tests were performed to locate the tumor; Hormonal assays (serotonin, insulin, gastrin, etc.)	Imaging studies (CT, MRI, positron emission tomography scans) and biopsies are to confirm the presence of the tumor and assess its characteristics; Biomarkers such as chromogranin A and others were assessed, but the elevation is not as prominent as in functional NENs
Treatment	Designed to control hormone secretion and manage associated symptoms; Surgical resection, targeted therapy, and SSAs may be used based on the type and stage of the tumors	If feasible, the focus is on surgical resection and may also include chemotherapy, targeted therapy, and SSAs; Prognosis usually depends on the stage and grade of the tumor, as well as the response to treatment

*CT* computed tomography, *MRI* magnetic resonance imaging, *NENs* neuroendocrine neoplasms, *SSAs* somatostatin analogs

## Pathology and grading system

The histological features of GEP-NENs corroborate their anatomical site and endocrine cell origin. GEP-NENs are characterized by the loss of epithelial tubular gland structures and relatively diffuse expression of neuroendocrine markers such as chromogranin A (CgA) [24]. The nuclear protein Ki-67 is expressed during the active phases of the cell cycle and plays a crucial role in dispersing mitotic chromosomes [25]. Therefore, the current classification systems proposed by the World Health Organization (WHO) and European Neuroendocrine Tumor Society (ENETS) are based on the Ki-67 index and cytological analyses of mitoses in histological material (Table 2) [5, 6]. Several clinical studies have shown that patients with grade 3 tumors exhibit significantly lower survival rates than those with grade 1 or grade 2 tumors. Furthermore, patients at the grade 2 stage experience notably worse survival outcomes than those at the grade 1 stage [1, 2, 4].

**Table 2:** WHO classification and grading criteria for GEP-NENs

Classification	Differentiation	Grade	Mitotic rate (mitoses/2 mm^2^)	Ki-67 index
NETs	Well-differentiated	Grade 1	< 2	< 3%
		Grade 2	2 – 20	3 – 20%
		Grade 3	> 20	> 20%
NECs	Poorly-differentiated	Grade 3	> 20	> 20%

*WHO* World Health Organization, *GEP-NENs* gastroenteropancreatic neuroendocrine neoplasms, *NET* neuroendocrine tumor, *NEC* neuroendocrine carcinoma

Recently, several novel classifications have been proposed that may be considered superior to the current classification for predicting malignant tumor biology and patient prognosis. For example, La Rosa et al. [26] proposed a new global histological grading system based on the WHO 2000 and ENETS-WHO 2010 grading systems combined with histological features to improve tumor prognostic stratification (grade 1 vs. grade 2, *P* = 0.007; grade 1 vs. grade 3, *P* < 0.001; grade 2 vs. grade 3, *P* = 0.001). The current classifications, although no longer reliant on anatomical location or histology, thereby reducing the inconsistencies of GEP-NEN diagnosis, still possess certain limitations. As previously indicated, NETs are classified into three grades, namely, grade 1, grade 2, or grade 3, which are determined based on the mitotic count and/or Ki-67 labeling index. To remove any ambiguity in the classification (2 – 3%), the grade cutoffs were gradually adjusted from grade 1 to grade 2 [27]. Furthermore, there has been a recent focus on the assessment of Ki-67 levels, as the reliability of these methods varies among different approaches. These findings suggest that the grade of NETs for the same patient may vary across different hospitals.

## Biological profiles and genetic differences in neuroendocrine tumors (NETs)

The origin of neuroendocrine cells can be traced back to gastrointestinal stem cells rather than neurocrest cells. In recent years, extensive efforts have been made to elucidate the biology of NETs. The protein synthesis, hormone secretion, and proliferation of these tumors primarily rely on interactions between somatostatin receptors (SSTRs) and their associated molecules. The induction of angiogenesis, survival, and metabolic acceleration in NENs depends on the upregulation of the mammalian target of rapamycin (mTOR). Proangiogenic factors, including platelet-derived growth factor, vascular endothelial growth factor, angiopoietin, semaphorins, and fibroblast growth factor, are involved in the tumorigenesis of NETs [28]. The tumor microenvironment, which consists of the extracellular matrix and various cellular components, such as stromal, inflammatory, and endothelial cells, plays a crucial role in influencing the biological behavior, proliferation, response to therapy, and propensity for developing fibrotic complications of tumors [29]. The degradation of the extracellular matrix can accelerate the carcinogenesis and progression of NETs. Moreover, NETs can secrete and utilize a diverse range of mediators, including platelet-derived growth factor and serotonin, to stimulate and enhance the proliferation of fibroblasts, ultimately leading to fibrosis. Furthermore, the upregulation of hypoxia-inducible factor 1-α can induces the secretion of proangiogenic factors, thereby accounting for the heightened vascular density observed in NETs [30]. The infiltration of immune-related cells can also establish an immunosuppressive microenvironment for the progression of NETs [31].

In addition to their distinct biological characteristics, NETs have a unique genetic profile. The genomic, epigenomic, and transcriptomic profiles of GEP-NETs vary based on the primary site and degree of differentiation. Genomic deletion is more prevalent in pancreatic NETs (pNETs) than chromosomal gain. Somatic mutations in multiple endocrine neoplasia type 1 and death domain associated protein/α-thalassemia, mental retardation, X-linked have been identified in 44% and 43% of pNETs, respectively, while 14% of tumor samples exhibited mutations in mTOR and related pathways, including phosphatase and tensin homolog, tuberous sclerosis complex 2, phosphatidylinositol-4,5-bisphosphate 3-kinase catalytic subunit alpha [32]. An investigation involving 102 primary pNETs demonstrated that 4 principal pathways, including DNA damage repair, chromatin remodeling, telomere maintenance, and mTOR induction, were aberrantly activated in cancers. Additionally, germline mutations have been observed in clinically sporadic pNETs, with genetic mutations identified in *MUTYH*, *CHEK2*, and *BRCA2* [33]. Compared with pNETs, insulinomas present distinct characteristics. Mutations in the *YY1* gene have been detected in 30% of samples from a cohort of 113 Asian patients with insulinoma [34]. However, mutations and genetic alterations are less common in gastrointestinal NETs than in pNETs. It has been observed that 60 – 90% of small bowel NETs lack chromosome 18, but these chromosomal changes do not result in significant biological effects [35]. In intestinal NETs, there is a low mutational rate, with *CDKN1B* gene mutations or deletions identified in only 8% of patients [36, 37]. However, it should be noted that *CDKN1B* mutation is not associated with disease progression, clinical course, or prognosis but is linked to heterogeneity within and between tumors [38]. In addition, small bowel carcinoids exhibit widespread DNA hypomethylation, and clinically aggressive behavior may be linked to a high methylation index [39]. The presence of substantial DNA methylation alterations in primary and metastatic tumors confirms the potential function of epigenetic dysregulation in the malignancy of small bowel NETs [40].

## Diagnosis of GEP-NENs

### Biomarkers

Numerous studies and guidelines have demonstrated that the serum concentration of CgA serves as a reliable biomarker for the detection of GEP-NENs [24, 41–45]. Notably, elevated levels of CgA have also been observed in several non-neuroendocrine carcinomas (NECs), such as lung, breast, and prostate carcinomas [46]. Moreover, multiple studies have proposed that diagnostic models incorporating CgA and other factors are more effective in diagnosing GEP-NENs than those relying solely on CgA. For example, it has been reported that the combined utilization of CgA and SSTR scintigraphy yields notably higher sensitivity and specificity than the use of CgA in isolation [47].

Recently, a range of novel biomarkers have been evaluated and demonstrated potential as future therapeutic tools for the management of NENs (Table 3) [48–64]. Kidess et al. [65] investigated the diagnostic value of secreted phosphoprotein 1 (SPP1) in NEN. This study revealed a significant correlation between SPP1 and the grade of NENs, while no correlation was found between CgA and SPP1. These results suggested that SPP1 can be utilized independently of CgA in the diagnostic assessment of NENs. Furthermore, CgA, rather than SPP1, serves as an indicator of tumor aggressiveness. Therefore, the combined use of CgA (a broad indicator of neuroendocrine cell activity and tumor burden) and SPP1 (associated with aggressive tumor behavior and metastasis) may provide additional benefits for the diagnosis of GEP-NENs. Additionally, a notably greater level of SPP1 was detected in patients with grade 3 NENs than in those with grade 1 or 2 NENs, with elevated baseline SPP1 levels predicting an unfavorable outcome characterized by poor progression-free survival (PFS). Thus, curative resection is recommended for these patients. These findings underscore the potential of SPP1 as a promising biomarker with significant diagnostic utility in GEP-NENs, as well as its potential role as a predictive tool for distinguishing grade 3 tumors from grade 1 or 2 tumors, thereby informing surgical decisions.

**Table 3:** New biomarkers for the diagnosis of gastroenteropancreatic neuroendocrine neoplasms (GEP-NENs)

Name	Description	NENs	References
PNMA2	The PNMA2 can be utilized as a biomarker for detecting the recurrence and relapse of small intestine neuroendocrine tumors (SI-NETs); The low levels of Ma2 autoantibody did not show progression and recurrence-free survival; The higher levels of Ma2 antibodies were detected in the blood samples of TLC and ALC patients compared with healthy patients	Gastrointestinal neuroendocrine carcinomas (NECs); SI-NETs	[48, 49]
SERPINA10	The poor expression of PMP22 and upregulation of SERPINA10 and SYT13 can cause invasion and metastasis of tumor cells	SI-NETs	[50]
SMARCA4, MLH1, TSC1, ATRX, and ATR	They are major molecular-related pathways that determine the cytolytic activity	GFP-NETs	[50, 51]
GRIA2, GPR112, OR51E1, CXCL14 and NKX2-3	GRIA2 shows specific expression by the neuroendocrine carcinoma cells; GPR112 and OR51E1 have the ability to encode proteins with plasma membrane association and are targets for the diagnosis and treatment of cancer. Moreover, OR51E1 demonstrates no mutation in the SI-NEC patients in the various stages of progression; CXCL14 and NKX2-3 demonstrate poor expression in liver metastases compared to the primary tumors; The general expression of OR51E1 and OMP is found in SI-NETs	Gastrointestinal NECs; SI-NECs	[48, 52, 53]
DcR3, TFF3 and Midkine	Utilization as biomarkers in cancer diagnosis and their expression is observed in the tumor samples DcR3 shows overexpression in stage IV of the disease; Upregulation of DcR3 and TFF3 mediates the unfavorable prognosis; High serum levels of DcR3, TFF3, and Midkine are found in SI-NET patients	SI-NETs	[54]
UCH-L1 and α-internexin	Demethylation of UCH-L1 promoter can increase its expression in tumor samples; The upregulation of UCH-L1 along with α-internexin provides better prognosis and survival of patients	Pancreatic NETs	[55]
Pancreatic polypeptide (PP) and CgA	Elevation in the serum levels of CgA (normal < 98 μg/L) and PP (normal < 100 pmol/L) has been found in 69% and 31% of patients, respectively; In the metastatic disease, the upregulation of PP and CgA was observed; CgA sensitivity for functioning, non-functioning, pancreatic and gastrointestinal tumors is 96%, 75%, 74%, and 91%, respectively; PP sensitivity for functioning, non-functioning, pancreatic, and gastrointestinal tumors is 54%, 57%, 63%, and 53%, respectively	GEP-NETs	[56, 57]
Circulating tumor DNA (ctDNA)	ctDNA was enriched in 30% of NEN cfDNA and 44% of patients possess at least one ctDNA-positive sample; The CNAs in the cfDNA were sensitive and specific for NENs; The ctDNA evaluation can be utilized for understanding the diagnosis and disease progression pattern	NENs	[58, 59]
PD-L1	PD-L1 and PD-1 demonstrated expression in 6% and 1% of tumor samples, respectively and 8% of peritumoral tissue samples demonstrated expression for both of these biomarkers; The expression of PD-1 causes the metastasis of the tumor during diagnosis; The presence of circulating PD-1^+^ PBMCs caused the progressive disease; The circulating PD-1^+^ PBMCs increased the expression of PD-L1 in the tumor cells	GEP-NETs	[60]
INSM1	All the primary GEP-NENs and 94% of metastatic GEP-NENs demonstrate the upregulation of INSM1; The sensitivity of INSM1 was similar to SYN and it showed higher sensitivity compared with CgA in primary and metastatic neoplasms; The specificity of INSM1 was comparable to CgA and it had higher specificity than SYN	Primary and metastatic NENs of the gastrointestinal and pancreaticobiliary tracts	[61, 62]
DLL3	DLL3 demonstrates expression in the 5 well-differentiated GEP-NETs; DLL3 was present in 76.9% of poorly-differentiated NECs; The upregulation of DLL3 mediates RB1-loss and, causes the poor clinical outcome	GEP-NENs	[63]
miR-7-5p	The overexpression of miR-7-5p is observed in tumor samples; The sera of all SI-NEN patients demonstrated overexpression of miR-7-5p; There was no association with the age, gender, and tumor stage	NENs of the small intestine	[64]

*PNMA2* paraneoplastic antigen Ma2, *SERPINA10* serpin family A member 10, *SMARCA4* SWI/SNF related, matrix associated, actin-dependent regulator of chromatin, subfamily a, member 4, *MLH1* mutL homolog 1, *TSC1* TSC complex subunit 1, *ATRX* ATRX chromatin remodeler, *ATR* ATR serine/threonine kinase, *GRIA2* glutamate ionotropic receptor AMPA type subunit 2, *ADGRG4* (GPR112) adhesion G protein-coupled receptor G4, *OR51E1* olfactory receptor family 51 subfamily E member 1, *CXCL14* C-X-C motif chemokine ligand 14, *NKX2-3* NK2 homeobox 3, *DcR3* decoy receptor 3, *TFF3* trefoil factor 3, *mdk. S* (MIDKINE) midkine S homeolog, *UCHL1* ubiquitin C-terminal hydrolase L1, *CgA* chromogranin A *INSM1* INSM transcriptional repressor 1, *DLL3* delta-like canonical Notch ligand 3, *mir-7* (miR-7-5p) miR-7 stem-loop, *NET* neuroendocrine tumor, *NEC* neuroendocrine carcinoma, *NEN* neuroendocrine neoplasm, *SI-NEC* small intestine neuroendocrine carcinoma, *SI*-*NEN* small intestine neuroendocrine neoplasm, *TLC* typical lung carcinoids, *ALC* atypical lung carcinoids, *PMP22* peripheral myelin protein 22, *SYT13* synaptotagmin-13, *OMP* olfactory marker protein, *cfDNA* circulating free DNA, *CNAs* copy number alterations, *PD-1* programmed cell death protein-1, *PD-L1* programmed death-ligand 1, *PBMCs* peripheral blood mononuclear cells, *SYN* synaptophysin

Moreover, advanced technologies such as “omics” analyses offer enhanced opportunities for the diagnosis of GEP-NENs. The NETest is a liquid biopsy test for NENs that improves the accuracy of cancer molecular diagnosis by detecting NET-associated genes, such as *Ki-67*, *SSTR1,* and *SSTR2* expression levels through reverse transcription polymerase chain reaction [66, 67]. Currently, 5 experiments have evaluated the diagnostic value of NETs in GEP-NENs and reported them to be surrogate biomarkers for CgA in the diagnosis and screening of NENs [67–71]. In addition to liquid biopsy technology, biosensors are another promising approach for accessing GEP-NENs. To this end, an electrochemical immunosensor was developed for the clinical detection of Ki-67. The established method can detect Ki-67 within the range of 4.0 – 800 pg/ml, with a remarkably low detection limit as low as 1.7 pg/ml. To assess practical feasibility, the immunosensor was compared with the conventional immunohistochemical staining method in the analysis of 8 rabbit tumor model samples. A minor discrepancy of less than 2.51% was observed, indicating a high level of concordance between the two methodologies [72]. Nonetheless, the real-time assessment of tumors was not deemed feasible using this approach. Further investigations focusing on innovative biosensor designs have the potential to establish correlations between Ki-67 levels, thereby offering valuable insights for clinical applications. Furthermore, an extended-gated organic field-effect transistor-based immunosensor was devised for the identification of CgA. This method enabled the detection of CgA in artificial saliva samples with a detection threshold of 0.11 µg/ml, showing promise for future clinical applications in cancer patients [73]. Moreover, surface-enhanced Raman spectroscopy (SERS) utilizing an anti-CgA antibody-capturing probe was employed for CgA analysis. The CgA-SERS probe technique yielded results comparable to those of Western blotting and superior outcomes compared to those of traditional immunohistochemistry [74].

### Endoscopy

Endoscopy has emerged as a promising strategy for the diagnosis of gastric, duodenal, and colorectal NENs, and it can detect asymptomatic early-stage gastrointestinal tract NENs [75, 76]. James et al. [77] reported that endoscopic ultrasound (EUS) could detect 26% of pNETs, while the results obtained from CT and other radiologic examinations were negative.

EUS-fine needle aspiration (EUS-FNA) was performed based on the principles of EUS. This strategy provides histological and cytological information on the lesions and helps medical professionals develop personalized treatments for these patients [78]. Despite its utility, EUS-FNA encounters challenges such as inadequate core tissue acquisition and sampling limitations. To overcome these limitations, EUS-fine needle biopsy (EUS-FNB) has been developed, in which cutting needles are used to obtain core samples and enhance diagnostic accuracy, as supported by various studies [78, 79].

Compared to traditional endoscopy, video capsule endoscopy or wireless video endoscopy is a novel and non-invasive procedure for diagnosing gastrointestinal tract carcinomas. This approach involves swallowing a capsule-sized camera, allowing visualization of the small intestine with a light source, a capability not feasible with traditional endoscopy. However, routine capsule endoscopy is limited and is primarily recommended for identifying causes of small intestine bleeding.

### Imaging

Although the endoscopic procedure has a very high sensitivity for diagnosing NETs, it also depends on the operator. Compared with morphological examination, endoscopy can detect only local lesions. Therefore, morphological examination using CT and MRI has been performed to assess the location and extent of GEP-NENs [80]. It has been reported that the diagnostic values of CT and MRI for the detection of primary neoplasms are similar; however, CT provides better spatial resolution and is an effective technique for the diagnosis of small bowel NETs [80, 81]. Therefore, the combination of CT and MRI is expected to provide combined benefits [82].

Most GEP-NENs express SSTRs [83, 84]. This allows for the detection of tumors by molecular imaging of SSTRs via radionuclide-labeled somatostatin analogs (SSAs), single-photon emission computed tomography (SPECT), or positron emission tomography (PET). The commonly used radionuclides in SPECT imaging are ^99m^Tc, ^131^I, and ^111^In, and the positron nuclides used in PET are ^18^F and ^68^Ga. To date, ^68^Ga-DOTA-DSA PET/CTs, such as ^68^Ga-DOTATATE, ^68^Ga-DOTANOC, and ^68^Ga-DOTATOC PET/CTs, have been developed to diagnose GEP-NENs [85]. Several studies have proven that ^68^Ga-DOTATATE PET/CT has high accuracy and has become the preferred strategy for diagnosing GEP-NENs [81, 86–89]. In addition, ^68^Ga-DOTA-DSA PET/CT was used to determine whether the uptake of radiotracers correlated with the response to peptide receptor radionuclide therapy (PRRT), indicating high potential in the treatment of advanced GEP-NENs [90].

^18^F-FDG PET/CT typically exhibits high accuracy in diagnosing aggressive tumors. However, the value of ^18^F-FDG PET/CT in the diagnosis of GEP-NENs is still controversial since GEP-NENs generally exhibit indolent biological behavior with low glycolytic activity. Several studies have reported that ^18^F-FDG PET/CT has an acceptable diagnostic value for aggressive GEP-NENs with a Ki-67 index greater than or equal to 10% and low expression of SSTRs [91, 92]. Recently, two imaging classifications of NENs were developed based on ^68^Ga-DOTATATE and ^18^F-FDG PET/CTs [93, 94]. Both of these classifications are superior to histological grade for predicting patient survival. These findings suggested that ^68^Ga-DOTATATE PET/CT combined with ^18^F-FDG PET/CT could improve the accuracy of diagnosing GEP-NENs. However, this combinational diagnostic strategy will increase the cost to patients, and the benefits still need to be evaluated in future studies.

### Artificial intelligence (AI) diagnostic models

AI is a new field of science that provides human cognitive abilities to perform complex tasks, such as decision-making, which can be performed only by humans. The emergence of AI has enabled the introduction of more accurate diagnostic models for determining patient prognoses and guiding clinical decision-making [95–101]. Bevilacqua et al. [95] developed a noninvasive model based on presurgical ^68^Ga-DOTANOC PET/CT and conventional diagnostic methods. It was observed that this model can accurately predict grade 1 or grade 2 primary pNETs, providing valuable clinical insights. Although surgical resection is the only therapeutic modality for treating pNETs, it can cause significant postoperative complications and mortality. Compared to pancreatic ductal adenocarcinoma and other malignant diseases, pNETs are indolent tumors. Surgical resection did not significantly increase the survival time of patients with grade 1 tumors, which are smaller than 1 cm in length. Therefore, with the support of the Bevilacqua model and other diagnostic models [95, 96, 98, 100], clinicians can predict tumor grade and select appropriate personalized treatments, follow-up regimens, or surgical resection methods for low-grade pNETs. Notably, there are several limitations of these studies. One of the most relevant drawbacks is that these studies were retrospective and had small populations. Thus, the value of these models should be evaluated in further larger prospective cohort studies.

## Management of GEP-NENs

### Active surveillance or endoscopic resection

Patients with small, well-differentiated, and asymptomatic GEP-NENs positioned in the stomach, duodenum, pancreas, and colorectum can be treated conservatively, such as through active surveillance or endoscopic resection. Numerous studies have addressed the benefits of conservative treatments for GEP-NENs [44, 102, 103]. However, due to the poor prognosis of small bowel NENs and the location of the appendix, conservative treatment is not recommended for tumors originating from these sites.

### Surgical resection

**Fig. 1:**
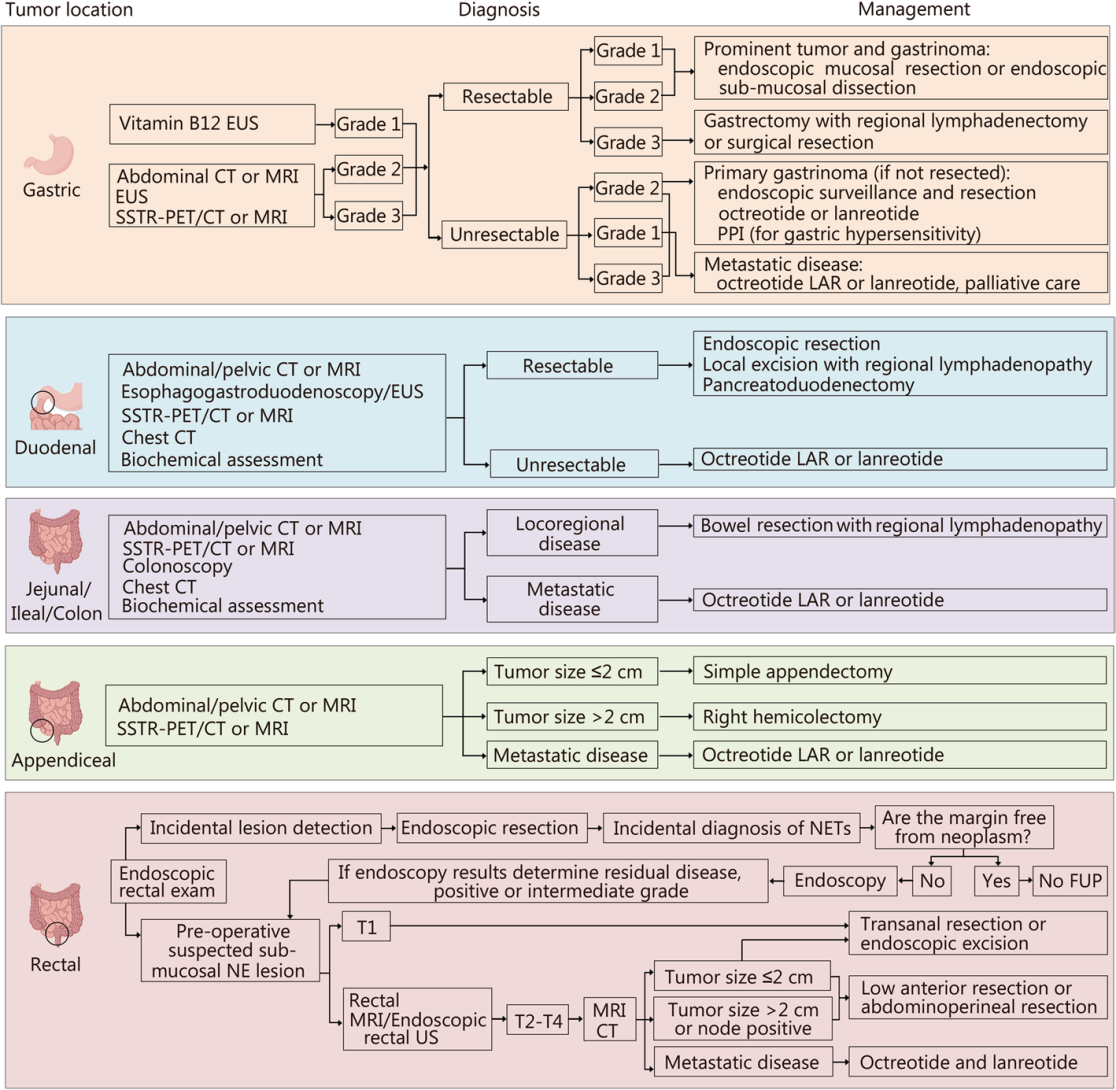
The diagnosis and management of GI-NENs. In different locations of the gastrointestinal tract, including the stomach, colon, and rectum, among others, tumors have been observed. Different strategies can be utilized to diagnose these tumors, including biochemical characteristics, MRI, biopsy, and endoscopy. According to the size of the tumor and grade, surgical resection can be recommended. Hence, the management of these tumors differs based on their origin, tumor size, and tumor grade. EUS endoscopic ultrasound, US ultrasound, CT computed tomography, MRI magnetic resonance imaging, SSTR-PET/CT somatostatin receptor-positron emission tomography/computed tomography, NE neuroendocrine, FUP follow-up, PPI proton pump inhibitor, LAR long-acting release, PRRT peptide receptor radionuclide therapy, T tumor, NETs neuroendocrine tumors, GI-NENs gastrointestinal neuroendocrine neoplasms

**Fig. 2:**
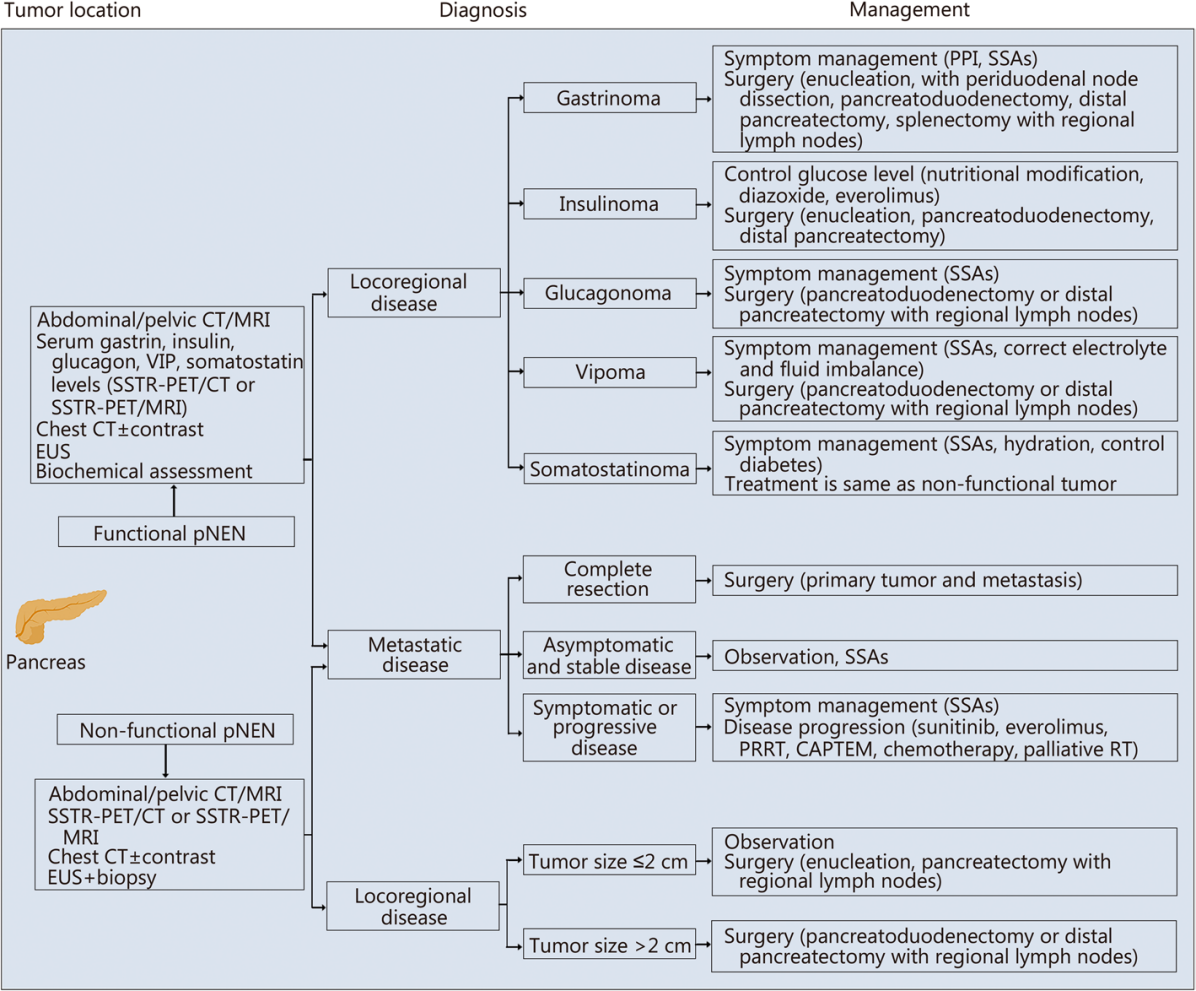
The diagnosis and management of pNENs. The management of functional and non-functional pNENs differs. However, similar examinations, including MRI, chest CT, and biopsy, can be performed for the diagnosis of these tumors. Disease management depends on the disease type, tumor size, and functional or non-functional status. EUS endoscopic ultrasound, US ultrasound, CT computed tomography, MRI magnetic resonance imaging, SSTR-PET/CT somatostatin receptor-positron emission tomography/computed tomography, PPI proton pump inhibitor, LAR long-acting release, pNEN pancreatic neuroendocrine neoplasm, SSAs somatostatin analogs, RT radiotherapy, PRRT peptide receptor radionuclide therapy, CAPTEM capecitabine and temozolomide, VIP vasoactive intestinal polypeptide

Surgical resection is the cornerstone for treating localized GEP-NENs. The choice of surgical technique, whether radical or palliative, depends on the specific location of the primary tumor (Figs. 1 and 2). In cases of grade 1 or grade 2 GEP-NENs with resectable or potentially resectable liver metastases, curative resection should be considered [104, 105]. Palliative resection of the primary tumor could be considered for unresectable metastases or for relieving symptoms of hormonal hypersecretion. Furthermore, for local treatment of liver metastases, radiofrequency ablation or hepatic artery embolization are also included as options in addition to surgical resection. The selection of these interventions should be based on the operator’s experience, the scope and location of liver metastases, and the blood supply to the metastases. The decision between radical and palliative surgical approaches is influenced by the location of the primary tumor [106].

### Medical treatment for advanced GEP-NENs

#### SSAs

SSAs can be used to control disease symptoms in NEN patients by inhibiting the overproduction of hormones and tumor growth. There are three main SSAs used in NEN treatment, namely, octreotide, lanreotide, and pasireotide. SSAs compete with somatostatin for binding to SSTRs and relieving symptoms such as diarrhea and flushing related to hormone secretion. Therefore, determining the expression of SSTRs by imaging is required before SSA therapy. Hence, the half-life of somatostatin is only several minutes, and long-acting SSAs, such as octreotide long-acting release (LAR), are widely used. However, several challenges still need to be addressed. The biological activity of the octreotide LAR fades over time, and supplemental therapy with subcutaneous immediate-release octreotide is needed. Moreover, some studies have reported that SSAs can lead to adverse effects, such as nausea, abdominal pain, and flatulence [107, 108]. These complications must be treated at the same time as the tumor.

The CLARINET trial indicated that lanreotide significantly prolonged PFS compared with placebo in patients with grade 1 or grade 2 metastatic GEP-NENs [109]. Therefore, lanreotide was approved in 2014 by the United States Food and Drug Administration (FDA) and is used to treat GEP-NENs. Recent European and United States guidelines state that increasing the dose of SSA by shortening the dosing interval may be an effective strategy for controlling the progression of tumors, and this finding is supported by the CLARINET FORTE phase II study, which proves that increasing the frequency of treatment is a valuable strategy before using less well-tolerated therapies for treating GEP-NENs [110].

The key distinction between SSA treatment protocols lies in their formulations and dosing regimens. Octreotide and lanreotide can be administered in both short- and long-acting formulations. Short-acting forms are used to manage symptoms immediately, whereas long-acting forms provide sustained effects over weeks. Octreotide LAR is typically given every 4 weeks, while lanreotide autogel is administered every 4 – 6 weeks, allowing for extended symptom control and tumor growth inhibition.

Pasireotide is different from octreotide and lanreotide because it is administered twice daily, which provides a continuous blockade of SSTRs. This difference in dosing frequency can be considered when tailoring treatment to individual patient preferences and optimizing symptom management and treatment of NENs. The choice of SSA and regimen was determined based on the specific type of NEN, stage of disease, and patient preference. The details of the trials on SSAs are mentioned in Table 4.

**Table 4:** Summary of clinical trials on the effect of SSAs, PRRT, and targeted therapies on neuroendocrine neoplasms

Treatment regimen	Clinical trial identifier	Study design	Type of tumor	Results
Octreotide LAR (30 mg intramuscularly in monthly intervals)	NCT00171873	Placebo-controlled, double-blind, phase IIIB randomized study	Metastatic mid-gut NETs	Octreotide LAR significantly lengthens the time to disease progression compared with the placebo
Lanreotide autogel (120 mg every 28 d)	NCT00353496	Phase III, randomized, double-blind, placebo-controlled, multicenter core study (CLARINET)	Non-functioning entero-pancreatic endocrine tumor	Lanreotide was significantly associated with PFS than the control group
Lanreotide autogel (120 mg every 28 d)	NCT00842348	CLARINET OLE study to assess the long-term safety of treatment	Non-functioning entero-pancreatic endocrine tumor	New evidence was found on the long-term safety profile and sustained antitumor effects of lanreotide autogel/depot
Pasireotide (SOM230 LAR 30 mg and LAR 10 mg)	NCT01374906	A randomized, double-blind, multicenter, phase III study	Cushing’s disease	Pasireotide LAR was well tolerated
Pasireotide LAR vs. octreotide LAR	NCT00690430	A randomized, multicenter, phase III study	Symptomatic refractory-resistant carcinoid disease	Pasireotide had PFS 5 months longer than octreotide
Lanreotide and octreotide LAR	NCT03017690	An observational time and motion analysis	Advanced GEP-NETs	Lanreotide was associated with significant reductions in drug-delivery time than octreotide LAR
Lutetium[^177^Lu] oxodotreotide injection	NCT05894486	A single-center, single-arm phase II study	GEP-NENs	Not yet recruiting
Lutetium[^177^Lu] oxodotreotide injection	NCT05884255	Open-label phase III Study	Advanced GEP-NENs	Recruiting
^177^Lu-DOTATATE	NCT01578239	Phase III open-label randomized trial (NETTER-1)	Inoperable, progressive, well-differentiated (grade 1 or grade 2) NETs	Improved PFS and OS compared to octreotide alone. Significant tumor response and symptom relief
^177^Lu-octreotate peptide receptor radionuclide therapy (Lutate PRRT)^±^, capecitabine and temozolomide (CAPTEM) or CAPTEM alone	ACTRN12615000909527	Phase II open-label randomized parallel-group trial	Pancreas and midgut NETs	CAPTEM/PRRT achieved target PFS with greater OTRR in both pNETs and mNETs, but there was more haem toxicity in mNETs
^177^Lu-edotreotide arm or everolimus	NCT03049189	Randomized, phase III, open-label trial	Inoperable, progressive, SSTR^+^ GEP-NETs	Ongoing trial
^177^Lu DOTA^−0^-Tyr^3^-octreotate ^68^Ga-DOTANOC PET/CT	NCT04609592	Phase I study	GEP-NENs	Recruiting
Everolimus (10 mg/d or placebo)	NCT00510068	Randomized double-blind, phase III trial (RADIANT-3)	Advanced pNET	Everolimus was associated with a median longest OS of 44 months
Everolimus (10 mg/d plus best supportive care or placebo plus best supportive care)	NCT01524783	Randomized, double-blind, placebo-controlled, phase III trial (RADIANT- 4)	Progressive NET of gastrointestinal or lung origin	Median PFS was 11.0 months in the everolimus group and 3.9 months in the placebo group
Surufatinib (300 mg/d or placebo)	NCT02589821	A randomized, double-blind, placebo-controlled, multicenter phase III trial (SANET-p)	Advanced pNET	The median PFS was 10.9 months for surufatinib vs. 3.7 months for placebo
Surufatinib (300 mg/d or placebo)	NCT02588170	A randomized, double-blind, placebo-controlled, phase III study (SANET-ep)	Advanced extra pNETs	The median PFS was 9.2 months in the surufatinib group vs. 3.8 months in the placebo group

*SSAs* somatostatin analogs*, PRRT* peptide receptor radionuclide therapy*, NCT* national clinical trial*, NET neuroendocrine tumor, PFS* progression-free survival*, OS* overall survival*, OTRR* objective tumor response rate*, mNETs* mid-gut NETs*, pNETs* pancreatic NETs*, LAR* long-acting release*, GEP-NET* gastroenteropancreatic neuroendocrine tumor*, CAPTEM* capecitabine and temozolomide*, SSTR* somatostatin receptor*, NETTER-1* neuroendocrine tumor therapy*, RADIANT-4* RAD001 in advanced NETs 4*, CLARINET* controlled study of lanreotide antiproliferative response in NETs*, SANET-p* surufatinib in advanced pancreatic NETs*, SANET-ep* surufatinib in advanced extrapancreatic NETs*, OLE* open-label extension, ^*177*^*Lu-DOTATOC* ^177^lutetium-DOTA^0^-D-Phe^1^-Tyr^3^-octreotide, ^*68*^*Ga-DOTANOC PET/CT* ^68^Ga-DOTA-D-Phe^1^-Nal^3^-octreotide positron emission tomography/computed tomography, ^*177*^*Lu-DOTATATE* ^177^lutetium-DOTA^0^-Tyr^3^-octreotate*, Lutate* lutetium-octreotate*, SOM230 LAR* pasireotide long-acting release

#### PRRT

Like that of SSAs, the therapeutic effect of PRRT also depends on the expression of SSTRs. The radiolabeled SSAs bind to SSTRs and are then internalized by the cells. Subsequently, the radionuclide damages the DNA by emitting α or β radiation. ^177^Lu-DOTATATE has several advantages in its production. Compared with the octreotide LAR, ^177^Lu-DOTATATE markedly enhanced PFS [111]. Therefore, ^177^Lu-DOTATATE is a widely utilized therapeutic strategy for patients with untreatable or metastatic GEP-NENs. Furthermore, PRRT is considered a neoadjuvant therapy for patients with borderline resectable tumors [112]. Several international phase III randomized clinical trials and experts have confirmed that ^177^Lu-DOTATATE could also be considered as a potential therapeutic agent due to its effect on the cytoreduction of tumors, which is rare among other existing alternative treatments (Table 4) [113]. A newly conducted phase III trial reported that the median overall survival of patients treated with ^177^Lu-DOTATATE 7.4 GBq (200 mCi) was 48.0 months, while that of patients treated with high-dose long-acting octreotide was 36.3 months [114]. This might be because ^177^Lu-DOTATATE is a radiation therapy that specifically targets SSTR-positive tumor cells using a radioactive isotope to directly kill tumor cells. This approach can have a more potent impact on tumor cells than high-dose long-acting octreotide, which is a hormone-based therapy that primarily works by inhibiting hormone secretion and tumor growth. In addition to the effectiveness of ^225^Ac-DOTATATE-targeted alpha therapy (TAT), this approach holds promise as a potential treatment for patients who lack a response to ^177^Lu-DOTATATE therapy or who have completed the maximum prescribed cycles of ^177^Lu-DOTATATE treatment [115].

α-emitting radionuclides, such as ^212^Pb-DOTAMTATE, ^213^Bi-DOTATATE, and ^225^Ac-DOTATATE, were labeled with SSAs and radiolabeled with SSTR antagonists. The limited soft tissue penetration of α-emitters minimizes radiation exposure to healthy tissues, enabling PRRT to be administered on an outpatient basis, thus introducing a novel aspect to patient care. α-particle emitters are preferred to β-particle emitters owing to their potential and specificity in sterilizing tumor cells from self-irradiation with α-particle emitters, a result that is not possible to obtain with β-particle emitters. However, some issues, such as clinical indications, and predictive and prognostic markers, need to be addressed in the future [116].

#### Targeted therapies

Everolimus and sunitinib are the most common targeted therapeutics for NENs lacking expression of SSTRs. Everolimus is a specific suppressor of mTOR, which controls mammalian cell size by targeting ribosomal protein S6 kinase beta-1 and 4E-binding protein 1 [117]. Everolimus inhibits the activity of mTOR, thereby blocking the proliferation and growth of NENs. Currently, everolimus is recommended for treating advanced and progressive pancreatic NENs and grade 1 or grade 2 non-functional gastrointestinal NENs. This is supported by the findings of two international phase III randomized controlled trials, RADIANT-3 [118] and RADIANT-4 [119]. The RADIANT-3 trial (NCT00510068), which included patients with pNETs, showed that patients treated with everolimus had significantly better median PFS than those treated with placebo, with PFS of 11.0 months and 4.6 months, respectively [118]. The RADIANT-4 trial (NCT01524783), which was conducted on patients with well-differentiated, non-functional NETs of the lung or gastrointestinal tract, revealed that everolimus was significantly correlated with improved PFS [119].

Everolimus is the first targeted treatment with a significant antitumor effect and acceptable tolerability across a broad range of pancreatic and gastrointestinal tract NETs [119]. Notably, a previous study showed that mTOR inhibitors are involved in adverse events such as hyperglycemia and hypercholesterolemia. These adverse events led to a non-significant improvement in PFS [120]. Recently, a pooled analysis of RADIANT-3 and RADIANT-4 demonstrated that adverse events do not affect PFS [121]. However, these results should be interpreted with caution due to the low number of adverse events observed, and additional experiments are needed to verify these findings.

As a suppressor of tyrosine kinase receptors, sunitinib inhibits the growth and metastasis of carcinoma. Currently, it is approved for treating only progressive, locally advanced, or metastatic pancreatic NENs [122]. The efficacy of sunitinib in treating other carcinomas needs to be addressed by future studies. The updated guideline for the treatment of distant metastatic disease caused by NENs mentioned that everolimus and sunitinib have antiproliferative effects on progressive pNETs. However, everolimus and sunitinib are prescribed as first-line therapies if chemotherapy is not clinically needed or if SSA is not a good option and cannot be tolerated. This is due to the potential toxicity of these target agents [123].

Several studies have evaluated the anticancer function of other angiogenic suppressors for treating non-pancreatic NENs [124–126]. Surufatinib in advanced pancreatic NETs (SANET-p) is a randomized, double-blind, placebo-controlled, and phase III study that evaluated the antitumor effect of surufatinib in the treatment of progressive and advanced, well-differentiated pNETs [127]. Surufatinib significantly prolongs PFS and has acceptable adverse effects. In addition, the antitumor effect of surufatinib in the treatment of extra pancreatic NENs was evaluated in the SANET trial [128]. The authors reported that patients treated with surufatinib, a novel oral tyrosine kinase inhibitor targeting immune cells and angiogenesis had remarkably improved PFS compared with patients treated with a placebo. It was found to be a therapeutic option for patients with GEP and thymic and lung NETs [129].

#### Chemotherapy

Chemotherapy is used to control tumor growth, alleviate symptoms, and improve overall survival in patients with GEP-NENs. There are a variety of chemotherapeutic strategies available for treating GEP-NENs. For example, the combination of streptozotocin-mediated chemotherapy and novel targeted drugs is being investigated for the treatment of grade 1/grade 2 pNETs. In the case of grade 1 or grade 2 GEP-NENs, chemotherapy is recommended for tumors located in the pancreas, and streptozotocin combined with 5-fluorouracil or temozolomide is the most commonly used strategy [44, 45, 75]. Streptozotocin-mediated chemotherapy is typically advised for patients with a substantial tumor burden, with or without associated clinical symptoms, or those experiencing significant tumorigenesis within a 6- to 12-month duration. Although there are limited results regarding temozolomide chemotherapy, it could serve as an alternative to the streptozotocin/5-fluorouracil regimen in patients for whom the latter is unavailable for pNETs. Platinum-based chemotherapy is prescribed as a first-line therapy for grade 3 NENs [123]. In patients with metastatic pancreatic endocrine carcinomas, capecitabine combined with temozolomide yielded a high and durable response. However, with extra pancreatic NENs, chemotherapy should only be considered when other therapies fail [6].

Patients with advanced, metastatic, or unresectable grade 3 GEP-NENs mainly undergo chemotherapy. Unfortunately, there are no standard therapeutic strategies for these patients. Although platinum-based chemotherapy prolongs the survival time of patients with a Ki-67 index of less than 55%, tumors are less responsive to these regimens [130]. Interestingly, temozolomide increased the response of tumors treated with capecitabine [131]. These findings suggested that capecitabine plus temozolomide-based chemotherapy might be the optimal chemotherapeutic strategy for patients with a Ki-67 index lower than 55%. Etoposide in combination with cisplatin is recommended for patients with a Ki-67 index greater than 55% [123]. According to the National Cancer Control Network (NCCN) guidelines, a clinical trial of well-differentiated grade 3 resectable NETs with relatively high Ki-67 index (> 55%) and rapid growth is preferred. However, neoadjuvant chemotherapy can also be given, and the options include temozolomide ± capecitabine, oxaliplatin-based therapy (FOLFOX or CAPEOX), cisplatin/etoposide or carboplatin/etoposide. Temozolomide may have a greater effect on tumors arising from the pancreas [132]. A recently published phase II randomized trial (NCT01824875) [133] indicated that the median PFS was 14.4 months for temozolomide and 22.7 months for capecitabine/temozolomide. Additionally, the median overall survival times for temozolomide and capecitabine/temozolomide were 53.8 months and 58.7 months, respectively [133]. Hence, the combination of temozolomide and capecitabine significantly improved PFS compared with temozolomide alone for advanced pancreatic NENs. Moreover, the FOLFOX regimen, folinic acid (FOL) plus fluorouracil (F) and oxaliplatin (OX), and the FOLFIRI regimen, folinic acid (FOL) plus fluorouracil (F) and irinotecan (RI), are recommended after the failure of first-line chemotherapy. Due to the unsatisfactory therapeutic outcomes of individual chemotherapies, some combinatorial therapeutic approaches are being evaluated in clinical trials. A randomized phase II parallel-group study evaluating the antitumor activity is currently recruiting patients. This study utilized ^177^Lu-PRRT in combination with capecitabine to treat grade 1 or grade 2 patients with a Ki-67 index greater than 20% or grade 3 patients with a Ki-67 index lower than 50% (NCT02736448).

Recent advancements in chemotherapy regimens have shown promise in enhancing treatment outcomes. A significant breakthrough involves the integration of targeted therapies with chemotherapy, such as the utilization of everolimus and sunitinib. These targeted treatments have exhibited disease control and prolonged PFS by disrupting crucial signaling pathways responsible for tumor growth and angiogenesis. Additionally, PRRT utilizing radiolabeled SSAs has emerged as a promising option, particularly for non-resectable or metastatic GEP-NENs expressing SSTRs. The evolving landscape of chemotherapy for GEP-NENs underscores the importance of a multidisciplinary approach, tailored treatment plans, and continued research to further refine therapeutic strategies and improve patient outcomes.

#### Immunotherapy

Immunotherapy has emerged as a novel therapeutic option, and ongoing clinical trials are currently evaluating its efficacy in GEP-NENs. Genetic profiling also plays a crucial role in predicting immunotherapy response [134].

Cancer immunotherapy has made remarkable advancements in recent years, resulting in notable therapeutic benefits across various tumor categories. Current approaches in immunotherapy include immune checkpoint inhibitors/blockades (ICIs/ICBs) that have shown promise in preclinical studies and early-phase clinical trials. Furthermore, adoptive T-cell therapy is being explored as a potential avenue for GEP-NENs. The utilization of ICB targeting of programmed cell death protein-1 (PD-1) and its corresponding ligand, programmed death-ligand 1 (PD-L1), represents a highly promising approach aimed at restoring the immune response against tumors. The FDA has approved the treatment of multiple tumor types, including melanoma, non-small cell lung cancer, head and neck squamous cell carcinoma, and urothelial cancer. A previous study has suggested that poorly-differentiated gastrointestinal NENs, mismatch repair deficiency, or microsatellite instability are indications for ICB [135].

Surgical resection and systemic chemotherapy are commonly used for the treatment of local and non-metastatic GEP-NENs [136]. However, the challenge arises as patients are often diagnosed at advanced stages with metastasis, complicating the efficacy of surgical resection and chemotherapy. The 5-year survival rate upon diagnosis is 57% for patients with well-differentiated tumors and merely 5.2% for those with small cell tumors. Chemotherapy is recommended for well-differentiated GEP-NETs exhibiting a low to moderate proliferation rate. Nevertheless, the FDA has suggested employing immunotherapy regimens to improve the treatment outcomes of these tumors. Several types of tumors can increase the levels of PD-1, PD-L1, and cytotoxic T lymphocyte-associated antigen-4 (CTLA-4) proteins, which play a role in suppressing T cell function and enabling tumors to evade the immune system surveillance, thereby facilitating uncontrolled growth [137]. For example, in high-grade (grade 3) and aggressive tumors, PD-L1 is overexpressed [60, 138–140].

Therefore, the application of ICBs and antibodies targeting such molecules for tumor eradication has increased. The use of oncolytic viruses to infect and destroy tumor cells also presents a promising option. Cancer vaccines have been introduced to activate the immune system by inducing major histocompatibility complex-I signaling on antigen-presenting cells for presenting tumor-associated antigens (TAAs). TAAs used for stimulating the immune system can derive from various sources, including whole-cell tumor lysates, full-length tumor proteins, DNA vaccines, or recombinant tumor peptides. There are multiple reasons to employ immunotherapy in treating NETs since they involve the upregulation of pathways related to immune evasion. In addition, there are other features associated with immunotherapy for the treatment of NETs. ICBs can serve as the primary treatment strategy for well-differentiated grade 3 NETs, mixed neuroendocrine-non-NENs, and poorly-differentiated extrapulmonary NECs. Patients with advanced tumors having high mutational burden, microsatellite instability-high, or mismatch repair deficiency could be recognized through an FDA-approved analysis before receiving pembrolizumab as a PD-1 inhibitor if no other treatment options exist. Moreover, pembrolizumab can be utilized as a primary treatment for biologically desirable or locally advanced/metastatic grade 3 NETs whose prognosis is undesirable [141, 142]. Pembrolizumab is suggested for systemic therapy of extrapulmonary, locoregional, unresectable, or metastatic NECs/mixed neuroendocrine-non-neuroendocrine neoplasms [141–143]. Furthermore, dual ICB therapy using nivolumab combined with ipilimumab is suggested in cases where locally progressed or metastatic grade 3 NETs exhibit undesirable biological profiles [143, 144].

The current evaluation of clinical trials is primarily focused on investigating the efficacy of ICBs in NENs originating from various sources, such as the gastrointestinal tract, pancreas, and lung [145–148]. The results obtained from phase II KEYNOTE-158 research indicated that pembrolizumab, an anti-PD-1 drug, exhibits a modest level of effectiveness in treating advanced well-differentiated NETs. Among the 107 patients included in the study, only 3.7% demonstrated a positive response to the treatment as measured by the objective response rate (ORR) [146]. Furthermore, findings from a multicohort, phase I KEYNOTE-028 study conducted on patients with PD-L1-positive NETs revealed that pembrolizumab treatment yielded an ORR of 12.0% for those with carcinoid tumors and 6.3% for those with well- or moderately-differentiated pNETs [148]. Additionally, results from a phase II basket trial examining blockade therapy involving anti-CTLA-4 (ipilimumab) and anti-PD-1 (nivolumab) agents showed that patients diagnosed with high-grade NECs exhibited an ORR of 44% (8 out of 18 patients), whereas those with low/intermediate grade NETs had an ORR of 0% (0 out of 14 patients; *P* = 0.004) [145]. Moreover, a multi-center phase Ib trial involving 40 NEN patients demonstrated a comparable response rate between the poorly-differentiated NEC subgroup and well-differentiated NET subgroup when treated with anti-PD-1 therapy using toripalimab (ORR: 18.7% vs. 25.0%) [147]. However, the inclusion of NENs in prior and ongoing clinical trials has been limited due to their rare occurrence. Hence, it is premature to make any definitive conclusions regarding the effectiveness of ICB in T-NEN treatment. Nonetheless, the potential use of ICB in treating well-differentiated NETs continues to generate promising outcomes.

### Therapeutic diet

The nutritional status of NEN patients is also an important parameter to consider in disease management. Patients with NETs, particularly those of gastroenteropancreatic origin, are expected to have excessive production of gastrointestinal hormones, peptides, and amines. These elevated levels are associated with malabsorption, diarrhea, steatorrhea, and altered gastrointestinal motility. Furthermore, the surgical and medical management of NENs also involves alterations in gastrointestinal secretory, motor, and absorptive functions, leading to dietary and nutritional implications. Several studies have proposed both Mediterranean and ketogenic diets as possible nutritional therapies for patients with GEP-NENs [21, 149]. Ketogenic diets mimic glucose starvation conditions, which inhibit tumor growth by modulating multiple signaling pathways, such as the phosphoinositide 3-kinase (PI3K)/protein kinase B (Akt) pathway, AMP-activated protein kinase (AMPK) pathway and mTOR pathway [150]. Another study reported the role of vitamin D deficiency in the incidence of GEP-NENs [20]. The efficacy of vitamin D was linked to high tumor grade and disease progression. It was recommended to monitor 25(OH)D levels in these patients, and vitamin D supplementation was suggested for the management of GEP-NEN patients [19].

## Challenges and limitations

GEP-NENs are a diverse group of rare tumors that can occur in various locations throughout the gastrointestinal tract and pancreas. Although GEP-NENs are uncommon, they can also pose a significant threat to patients. Diagnosis and management of these disorders can be challenging due to several factors. (1) Limited knowledge and experience. GEP-NENs are relatively rare tumors, and many clinicians may not have experience in diagnosing and treating them. Hence, this may lead to delays in diagnosing these tumors, resulting in their detection at advanced stages when the tumors exhibit aggressive progression. (2) Limited understanding of the biology and prognosis. GEP-NENs are characterized by a high degree of genetic diversity and complexity, which poses challenges to understanding the underlying biology and developing targeted therapies. Furthermore, epigenetic alterations control other biological and molecular mechanisms of these tumors, but these changes require further investigation and clarification. In addition, the prognosis of GEP-NENs is unclear, and reliable prognostic markers are lacking. Future studies should also consider the role of extracellular vesicles as minimally invasive factors. (3) Insidious onset of symptoms. Because of their slow growth and slow progression, GEP-NNEs are often difficult to detect in the early stages. These patients are either asymptomatic in the early stages or have general symptoms similar to other diseases. (4) Limited availability of biomarkers. There are currently no specific biomarkers for diagnosing GEP-NENs, making it difficult to distinguish them from other types of tumors. (5) There is currently a lack of consensus on the classification and grading of GEP-NENs, which may lead to confusion and inconsistent management. (6) Treatment options for GEP-NENs are limited, and there is no standard of care for advanced disease.

## Conclusions and future perspectives

Notably, recent epidemiological studies have shown that the incidence of GEP-NENs has significantly increased in the last twenty years. However, as GEP-NENs are highly heterogeneous malignancies with indolent and aggressive tumor biology, they remain orphan diseases, and both their diagnosis and treatment have been neglected for a long time. Despite recent theoretical and clinical advances showing significant improvements in the diagnosis and treatment of GEP-NENs, clinical outcomes and survival results remain unsatisfactory [151]. Therefore, innovation is needed in the diagnosis, prognosis, and treatment of these tumors.

Genetics and molecular factors play crucial roles in explaining differences in incidence, pathophysiology, clinical signs, and treatment outcomes. Gender differences in cancer incidence are mainly attributed to genetic and molecular regulation, along with the influence of sex hormones on gene expression in various types of cancers, although little is known about the impact of these factors on GEP-NENs [152–154].

The WHO- and ENETS-based grading systems represent a milestone in the classification and nomenclature of NENs according to cytological and histological scores. The discordance between grades assessed by mitotic counting or the Ki-67 index is often contradictory, and grades are usually greater when determined by Ki-67. Recently, consistency in Ki-67 assessment has been improved by the use of AI microscopy [155]. We believe that with the advent of several technologies and the incorporation of new conceptual approaches, we can lay the groundwork for the next generation of NEN classifications that will make classifications more consistent in understanding how neoplasms from different organ systems are clinically and genetically related to each other [151, 155–157].

Regarding the treatment and management of GEP-NENs, conservative management is recommended for patients with small and asymptomatic GEP-NENs, whereas surgical resection is advised for those with localized tumors. However, considering the heterogeneity of GEP-NENs, the choice of surgical approach should be based on the location and clinicopathological characteristics of the tumor. Currently, only everolimus, sunitinib, ^177^Lu-DOTATATE, and PRRT are approved treatments for GEP-NENs; however, further evaluation is needed to determine the benefits of chemotherapy and immunotherapy.

In addition, both medical and surgical treatment of NENs can lead to alterations in gastrointestinal functions, with both dietary and nutritional implications. In this scenario, tailored nutritional approaches have been shown to alleviate multiple clinical conditions in NEN patients [158, 159]. It is highly relevant and useful to assess the effect of different clinical nutrition nursing on perioperative immune status, postoperative bowel motility, and complications in patients with GEP-NENs [160]. Notably, several studies have proposed both Mediterranean and ketogenic diets (with low-fat content, low carbohydrate content, natural unsaturated fat content, high antioxidant content, high chemopreventive phytochemical content, and high fiber content) as possible nutritional therapies for GEP-NEN patients [29, 149, 160]. Ketogenic diet creates a state similar to glucose deprivation where the body produces ketones to increase energy and ATP production. The ketogenic diet has demonstrated beneficial effects in cancer treatment by regulating insulin/insulin-like growth factor, PI3K/Akt/mTOR, and AMP-activated protein kinase to suppress cancer progression and proliferation [150]. Moreover, they can also serve as adjuvant therapies with conventional chemotherapy and radiation therapy.

Finally, to effectively manage patients, healthcare professionals and skilled nutritionists must adopt an approach based on multidisciplinary decision-making and possess a precise comprehension of information and communication. The clinicians’ understanding of the classification systems and the importance of novel markers is highly appreciated and recommended.

## Data Availability

Not applicable.

## Abbreviations

AI: Artificial intelligence
CgA: Chromogranin A
ENETS: European Neuroendocrine Tumor Society
EUS: Endoscopic ultrasound
EUS-FNA: EUS-fine needle aspiration
FDA: Food and Drug Administration
GEP-NENs: Gastroenteropancreatic neuroendocrine neoplasms
ICB: Immune checkpoint blockade
LAR: Long-acting release
MRI: Magnetic resonance imaging
mTOR: Mammalian target of rapamycin
NEC: Neuroendocrine carcinoma
NENs: Neuroendocrine neoplasms
NET: Neuroendocrine tumor
ORR: Objective response rate
PET: Positron emission tomography
PFS: Progression free survival
pNET: Pancreatic neuroendocrine tumor
PRRT: Peptide receptor radionuclide therapy
SPP1: Secreted phosphoprotein 1
SSAs: Somatostatin analogs
SSTRs: Somatostatin receptors
SERS: Surface-enhanced Raman spectroscopy
US: Ultrasound
WHO: World Health Organization
